# Prevalence and Risk Factors for Tuberculosis Infection among Hospital Workers in Hanoi, Viet Nam

**DOI:** 10.1371/journal.pone.0006798

**Published:** 2009-08-27

**Authors:** Luu Thi Lien, Nguyen Thi Le Hang, Nobuyuki Kobayashi, Hideki Yanai, Emiko Toyota, Shinsaku Sakurada, Pham Huu Thuong, Vu Cao Cuong, Akiko Nanri, Tetsuya Mizoue, Ikumi Matsushita, Nobuyuki Harada, Kazue Higuchi, Le Anh Tuan, Naoto Keicho

**Affiliations:** 1 Hanoi Tuberculosis and Lung Disease Hospital, Hanoi, Viet Nam; 2 International Medical Center of Japan - Bach Mai Hospital (IMCJ-BMH) Medical Collaboration Center, Hanoi, Viet Nam; 3 Department of Respiratory Medicine, Toyama Hospital, International Medical Center of Japan, Tokyo, Japan; 4 Institute of Tropical Medicine, Nagasaki University, Nagasaki, Japan; 5 Department of Respiratory Diseases, NHO Tokyo Hospital, Tokyo, Japan; 6 Department of Respiratory Diseases, Research Institute, International Medical Center of Japan, Tokyo, Japan; 7 General Planning Department, Hanoi Tuberculosis and Lung Disease Hospital, Hanoi, Viet Nam; 8 Department of Epidemiology and International Health, Research Institute, International Medical Center of Japan, Tokyo, Japan; 9 Department of Mycobacterium Reference and Research, Research Institute of Tuberculosis, Tokyo, Japan; 10 Hanoi Department of Health, Hanoi, Viet Nam; McGill University, Canada

## Abstract

**Background:**

Transmission of tuberculosis (TB) to health care workers (HCWs) is a global issue. Although effective infection control measures are expected to reduce nosocomial TB, HCWs' infection has not been assessed enough in TB high burden countries. We conducted a cross-sectional study to determine the prevalence of TB infection and its risk factors among HCWs in Hanoi, Viet Nam.

**Methodology/Principal Findings:**

A total of 300 HCWs including all staff members in a municipal TB referral hospital received an interferon-gamma release assay (IGRA), QuantiFERON-TB Gold In-Tube^TM^, followed by one- and two-step tuberculin skin test (TST) and a questionnaire-based interview. Agreement between the tests was evaluated by kappa statistics. Risk factors for TB infection were analyzed using a logistic regression model. Among the participants aged from 20 to 58 years (median = 40), prevalence of TB infection estimated by IGRA, one- and two-step TST was 47.3%, 61.1% and 66.3% respectively. Although the levels of overall agreement between IGRA and TST were moderate, the degree of agreement was low in the group with BCG history (kappa = 0.29). Working in TB hospital was associated with twofold increase in odds of TB infection estimated by IGRA. Increased age, low educational level and the high body mass index also demonstrated high odds ratios of IGRA positivity.

**Conclusions/Significance:**

Prevalence of TB infection estimated by either IGRA or TST is high among HCWs in the hospital environment for TB care in Viet Nam and an infection control program should be reinforced. In communities with heterogeneous history of BCG vaccination, IGRA seems to estimate TB infection more accurately than any other criteria using TST.

## Introduction

Transmission of *Mycobacterium tuberculosis* (MTB) in health care facilities is a problem worldwide [1]–[3]. Occupational tuberculosis (TB) can lead to the loss of skilled workers and impact health care service adversely, which has serious consequences in association with recent spread of multi-drug resistant (MDR) MTB strains [1]. Effective infection control measures are expected to reduce nosocomial TB [3]–[6]. In this sense, estimation of prevalence and risk for TB infection among health care workers (HCWs) involved in TB care is one of the essential steps to review and reinforce TB control measures.

In TB high burden countries, however, occupational risk for TB has often been neglected and concealed by the high prevalence in the general population. Furthermore, in those countries, widespread use of BCG vaccination has interfered with interpretation of tuberculin skin testing (TST) [1], [7], which was the only measure to detect TB infection until recently.

A newly developed diagnostic test designated as the interferon-gamma release assay (IGRA) uses a principle that MTB-specific antigens provoke immune reaction in the whole blood after TB infection [8]. With the advent of IGRA, many investigators have reported that latent TB infection could be detected more specifically than using TST [9]–[11]. QuantiFERON-TB Gold test, an ELISA-based IGRA, is also recommended by the US Centers for Disease Control and Prevention (CDC) for initial and sequential-testing of latent TB infection among HCWs [12].

Viet Nam is one of the 22 TB high burden countries defined by WHO, with prevalence of TB being 227/100,000 population and drug resistance TB is ever-increasing [13]. In Hanoi, the capital of Viet Nam, prevalence of smear positive pulmonary TB is 146/100,000 population [14] and the annual risk of TB infection reported from the suburban area is 0.8% [15]. Despite the high burden, little is known about TB infection among HCWs. We conducted this study to estimate the prevalence and risk factors for TB infection among HCWs in a crowded TB referral hospital together with an adjacent general hospital in Hanoi, Viet Nam, by comparing IGRA with conventional TST one- and two-step methods.

## Methods

### Ethics statement

A written informed consent was obtained from each participant. The study was approved by ethical committees of the Ministry of Health, Viet Nam and International Medical Center of Japan respectively.

### Study design and setting

**Figure 1:**
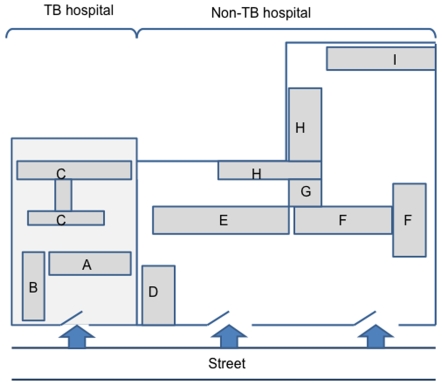
Allocation of two hospitals. TB: Tuberculosis. TB hospital buildings: A = Administration; B = Outpatient clinic and Laboratory; C = Wards and Imaging diagnosis. Non-TB hospital buildings: D = Emergency; E = Wards; F = Laboratory; G = Administration and Imaging diagnosis; H = Out-patient clinic and Wards; I = Imaging diagnosis.

We conducted a cross-sectional study in November 2007 in two hospitals adjacently located in the same block of Hai Ba Trung District in Hanoi city (Figure 1). A 110-bed “TB hospital”, which receives 2,000 TB in-patients and 46,000 turns of examination per year, is mainly assigned for taking care of TB patients in the entire city. The other is a 460-bed “non-TB hospital”, which is a general hospital but transfers all TB-suspected patients to the afore-mentioned TB hospital.

### Participants and data collection

Sample size was determined by the number of all staff members in the TB hospital, since our goal was to clarify the situation of all available HCWs working in the environment. The same categories of departments, such as outpatient clinic, intensive care unit, departments of internal medicine, laboratory and administration were selected from the non-TB hospital and the equivalent number was randomly extracted from each category. Demographic information, history of BCG vaccination and factors potentially associated with TB exposure were collected by an interview using a structured questionnaire. Those factors included job category, duration of working, practice of wearing mask, and professional or household contact with TB patients. For all participants, the blood was collected for IGRA, then TST was administered but not for those with pregnancy, breast-feeding or allergy to tuberculin. To rule out active TB, chest X-ray was taken for all participants with positive IGRA results. Sputum test was performed for participants with productive cough. They also had the chance of receiving INH for treatment of latent TB infection if they wished, after consultation with TB doctors there.

### TST and IGRA

As the first TST, a 5-tuberculin unit dose of Purified Protein Derivatives (Pasteur institute, Nha Trang, Viet Nam), authorized by the Ministry of Health of Viet Nam, was administered by well-trained technicians. Diameter of the induration size was measured after 48 to 72 hours, using a standardized ruler. If the size was less than 10 mm, the second administration with the same dosage was given after 14 days and the results were interpreted similarly (the second TST). From experience in Viet Nam [15], a cut-off value of 10 mm was used in this study, unless otherwise specified. Possible effects of changing cut-off values from 11 mm to 15 mm were also evaluated.

IGRA for TB is a method to measure interferon-gamma induced by MTB-specific antigens (TB antigen) to detect infection. In this study, the newest version of ELISA-based IGRA, QuantiFERON-TB Gold In-Tube^TM^ (Cellestis, Victoria, Australia), was used. One milliliter of the whole blood was collected separately in each heparin-containing tube pre-coated with nil for negative control, mitogen for positive control, and TB antigen. After 18-hour incubation in 37°C, each tube was centrifuged and plasma was harvested. Concentration of interferon-gamma in the plasma was measured using the ELISA method and calculated using analytical software recommended by the manufacturer. The cut-off value of interferon-gamma concentration was 0.35 IU/ml calculated from TB antigen minus negative control. Based on the algorithm of the software, the result was considered to be indeterminate in one of the following two conditions: the nil value itself was higher than 8.0 IU/ml, or mitogen minus nil value was less than 0.5 IU/ml in addition to TB antigen minus nil was less than 0.35 IU/ml. The testing procedure was carefully monitored [16] and quality control of the test was done in each run, following the manufacturer's instruction.

### Statistical analysis

To compare proportions in two groups, chi-squared test was used. Mantel-Haenszel method for stratified data was also attempted. Agreement between TST and IGRA was quantified using kappa statistic. Symmetry test equivalent to McNemar test was used to evaluate the symmetry of discordant results, TST+/IGRA- and TST-/IGRA+. To determine whether history of BCG vaccination or other factors interprets discordant results, unadjusted and adjusted odds ratios were calculated using a logistic regression model. The associations between potential risk factors and TB infection estimated by IGRA positivity were also evaluated by multivariate analysis using a logistic regression model, with IGRA result as outcome and factors possibly related to tuberculosis infection as independent variables. Biologically significant variables such as sex and other variables showing *p* values <0.20 in the univariate analysis were included in the multivariate model. All statistical analyses were performed using Stata version 10 (StataCorp, College Station, TX) and *p*<0.05 was considered to be statistically significant.

## Results

### Characteristics of study population

As shown in Table S1, a total of 300 HCWs of the two hospitals participated in our study and the majority of these were female. The median age was 40 years old, ranging from 20 to 58. Educational levels depended on job categories, but two thirds were at pre-university level or lower. More than one third of the participants had a history of BCG vaccination, of which more than 95% had actual BCG scar (data not shown).

Participants to the study included all of the 150 HCWs working in the TB hospital and 150 of 803 HCWs from the non-TB hospital (Table S1). Two thirds of HCWs in the TB hospital were less than 40 years old and this proportion was larger than in the non-TB hospital (*p*<0.0001, table not shown).

### Study flow

**Figure 2:**
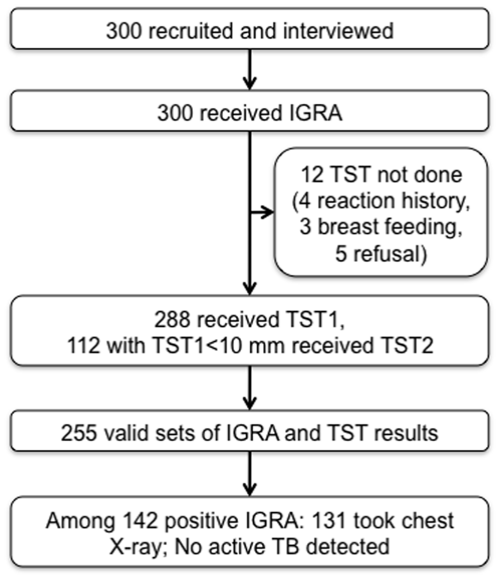
Study flow diagram. IGRA: Interferon-gamma release assay; TST1: The first Tuberculin skin test; TST2: The second Tuberculin skin test; TB: Tuberculosis.

As shown in Figure 2, all 300 participants provided blood for IGRA, while 288 of the 300 received TST. Out of them, 112 (38.9%) HCWs whose induration size of the first TST was less than 10 mm took the second TST. IGRA results were indeterminate in 35 (11.7%) individuals, in which 33 received TST and 2 did not. Since IGRA-TST data sets were analyzable when positive or negative results were obtained from both tests, these 33 IGRA indeterminate results were subtracted from 288 TST results, making 255 valid data sets. For check-up of active pulmonary TB, 131 of 142 IGRA-positive HCWs took chest radiography. Spontaneously cured tuberculosis was not completely excluded in 11 individuals (data not shown). Although active TB could not be ruled out from the chest radiography in one individual, all of the IGRA-positive individuals did not report any signs or symptoms in the follow-up period and were regarded as having latent TB infection. TST results were not emphasized in making this decision, because we expected that false-positive TST results due to previous BCG vaccination were not clinically negligible. None of them agreed to take INH treatment.

### TST and IGRA positivity

TST measurements were obtained in 288 individuals. With a cut-off value of 10 mm, 176 of 288 (61.1%) were positive after the initial injection as a result of conventional “one-step” TST (Table not shown). Of 112 participants with negative TST initially, 15 turned into positive after the second injection, increasing the overall positivity up to 66.3% as a result of “two-step” TST. When 15 mm of induration size was used as the cut-off value, positive results were decreased to half (29.5%). Positive or negative IGRA results were obtained in 265 out of 300 individuals (88.3%). With the cut-off point of IGRA described in the method section, 142 were positive out of the total 300 tested (47.3%).

Since age distribution was rather different between the two hospitals, data stratified by age of each hospital was shown in Table 1. In the TB hospital, positive TST results with one-step TST and 10-mm cut-off value accounted for 54.9%, 72.7%, 86.5% and 85.7% in groups of 20–29, 30–39, 40–49 and ≥50 years old respectively. IGRA results in the same hospital revealed positive in 38.2%, 47.9%, 51.3% and 87.5% for the corresponding age groups. The proportion of TST ≥10 mm was higher in the TB hospital than in the non-TB hospital by the Mantel-Haenszel test. IGRA positivity in the TB hospital had a similar tendency as compared with that in the non-TB hospital when stratified by age, although the difference did not reach statistical significance (Table 1).

**Table 1:** Proportion of IGRA and TST positivity, stratified by age.

		Non-TB hospital		TB hospital		*p* value
		No. positive/No. tested	(%)	No. positive/No. tested	(%)	
IGRA						
	20–29*	6/26	(23.1)	21/55	(38.2)	
	30–39	4/13	(30.8)	23/48	(47.9)	
	40–49	43/87	(49.4)	20/39	(51.3)	
	≥50	18/24	(75.0)	7/8	(87.5)	
	Combined**					0.13
One-step TST, ≥10 mm						
	20–29*	7/26	(26.9)	28/51	(54.9)	
	30–39	5/13	(38.5)	32/44	(72.7)	
	40–49	47/86	(54.7)	32/37	(86.5)	
	≥50	19/24	(79.2)	6/7	(85.7)	
	Combined**					<0.0001
One-step TST, ≥15 mm						
	20–29*	2/26	(7.7)	5/51	(9.8)	
	30–39	5/13	(38.5)	15/44	(34.1)	
	40–49	27/86	(31.4)	17/37	(45.9)	
	≥50	12/24	(50.0)	2/7	(28.6)	
	Combined**					0.45
Two-step TST, ≥10 mm						
	20–29*	8/26	(30.8)	28/51	(54.9)	
	30–39	5/13	(38.5)	33/44	(75.0)	
	40–49	60/86	(69.8)	32/37	(86.5)	
	≥50	19/24	(79.2)	6/7	(85.7)	
	Combined**					0.0004

TB: Tuberculosis; IGRA: Interferon-gamma release assay; TST: Tuberculin skin test.

* Years old.

** Mantel-Haenszel test for stratified data.

### Agreement between TST and IGRA

TST using different cut-off values were compared with IGRA in 255 sets of data (Table 2). Overall kappa values showed moderate agreement (kappa = 0.4 to 0.6) between TST and IGRA, whereas high cut-off values such as 13 mm and 15 mm of TST did not further increase the degree of agreement. As compared with one-step TST with the cut-off value of 10 mm (agreement rate = 72.5%, kappa = 0.44), two-step TST did not have any favorable effect on the degree of agreement (agreement rate = 71.0%, kappa = 0.41). In the group with BCG history, the degree of agreement was rather low, in contrast to the group without BCG history (kappa = 0.29 vs. 0.55) (Table 2).

**Table 2:** Agreement between IGRA and TST using different cut-off values of TST.

		One-step TST						Two-step TST
		≥10 (mm)*			≥11 (mm)*	≥13 (mm)*	≥15 (mm)*	≥10 (mm)*
			BCG (−)**	BCG (+)**				
TST+/IGRA+ (n)		114	44	39	102	86	69	119
TST+/IGRA- (n)		49	14	27	30	21	13	58
TST-/IGRA+ (n)		21	8	8	33	49	66	16
TST-/IGRA- (n)		71	33	23	90	99	107	62
Agreement, %		72.5	77.8	63.9	75.3	72.5	69.0	71.0
Kappa (SE)		0.44 (0.06)	0.55 (0.10)	0.29 (0.09)	0.50 (0.06)	0.46 (0.06)	0.39 (0.06)	0.41 (0.06)
Symmetry test***	Chi-squared value	11.2	1.64	10.3	0.14	11.2	35.6	23.8
	*p* value	0.0008	0.20	0.0013	0.71	0.0008	<0.0001	<0.0001

IGRA: Interferon-gamma release assay; TST: Tuberculin skin test; SE: Standard error; BCG (−): Without history of BCG vaccination; BCG (+): With history of BCG vaccination;

* N = 255; all subjects with valid data sets.

** N = 99 and 97 for BCG (−) and BCG (+) groups, respectively.

*** Equivalent to McNemar test for evaluation of the symmetry of TST+/IGRA- and TST-/IGRA+.

These findings prompted us to investigate the source of disagreement. With the cut-off value of 10 mm, the number of TST+/IGRA- individuals was disproportionately larger than that of TST-/IGRA+ individuals, which was statistically significant by the symmetry test (*p* = 0.0008). This disproportion was predominant in the subgroup with BCG history, but not in that without BCG history (*p* = 0.0013 vs. 0.20 respectively), when the same cut-off value was applied. Conversely, TST-/IGRA+, the other type of discordance, was strikingly increased when cut-off values of 13 or 15-mm were used (*p* = 0.0008 and p<0.0001 respectively) (Table 2).

Consequently, we investigated more deeply into factors associated with discordant results. In univariate analysis, BCG vaccination showed a significant association with TST+/IGRA- discordant results, with OR = 2.34 (95%CI, 1.14–4.81), when the other combinations were set as controls [10], [11]. In multivariate analysis, when age, working hospital and working duration were included in the model, BCG was the only parameter showing significant association with this discordance (OR = 2.26 [95%CI, 1.09–4.71]) (Table not shown). No factors analyzed in this study showed association with TST-/IGRA+ discordance.

### Factors associated with IGRA positivity

We tried to identify factors associated with IGRA positivity. In univariate analysis, non-occupational factors such as age and the high body mass index (BMI) were significantly associated with having a positive IGRA result (OR = 1.05 [95%CI, 1.02–1.07] per one year and OR = 5.10 [95%CI, 1.45–17.99], respectively) (Table 3), whereas occupational factors including job category, working duration, and mask use did not show significant associations with IGRA positivity (Table 3).

**Table 3:** Logistic regression analysis results for the associations between potential risk factors and IGRA positivity (n = 265).

		Proportion of positive results		Uni-variate		Multi-variate	
		n	(%)	Odds Ratio	(95%CI)	Odds Ratio	(95%CI)
Non-occupational factors:							
Age	/year	NA*	NA*	1.05	(1.02–1.07)	1.06	(1.00–1.11)
Sex	Female	102/197	(51.8)	1.00	(reference)	1.00	(reference)
	Male	40/68	(58.8)	1.33	(0.76–2.32)	1.10	(0.56–2.16)
BMI	18.5≤<25.0	114/223	(51.1)	1.00	(reference)	1.00	(reference)
	<18.5	12/23	(52.2)	1.04	(0.44–2.46)	1.50	(0.57–3.94)
	25.0≤	16/19	(84.2)	5.10	(1.45–17.99)	4.18	(1.14–15.36)
Education	University and higher	47/93	(50.5)	1.00	(reference)	1.00	(reference)
	High school and lower	25/36	(69.4)	2.22	(0.98–5.04)	4.28	(1.28–14.27)
	Pre-university	70/136	(51.5)	1.04	(0.61–1.76)	3.54	(1.18–10.59)
Occupational factors:							
Hospital	Non-TB	71/136	(52.2)	1.00	(reference)	1.00	(reference)
	TB	71/129	(55.0)	1.12	(0.69–1.82)	1.94	(1.04–3.64)
Job	Others	45/74	(60.8)	1.00	(reference)	1.00	(reference)
	Doctor	38/66	(57.6)	0.88	(0.45–1.72)	2.60	(0.82–8.29)
	Nurse	45/98	(45.9)	0.55	(0.30–1.01)	0.78	(0.28–2.19)
	Technician	14/27	(51.9)	0.69	(0.29–1.69)	1.02	(0.31–3.35)
Working years	<2	12/29	(41.4)	1.00	(reference)	1.00	(reference)
	2≤<5	20/47	(42.6)	1.05	(0.41–2.68)	0.94	(0.34–2.58)
	5≤<10	22/44	(50.0)	1.42	(0.55–3.65)	0.85	(0.28–2.56)
	10≤	88/145	(60.7)	2.19	(0.97–4.92)	0.91	(0.27–3.13)
Mask use	Frequently	65/124	(52.4)	1.00	(reference)	1.00	(reference)
	Occasionally	40/82	(48.8)	0.86	(0.50–1.51)	1.02	(0.55–1.88)
	Rarely/never	37/59	(62.7)	1.53	(0.81–2.88)	1.78	(0.81–3.94)

* NA = Not applicable.

In multivariate analysis, significantly increased odds of IGRA positivity were observed with non-occupational factors such as increase in age (OR = 1.06 [95%CI, 1.00–1.11]), high BMI (OR = 4.18 [95%CI, 1.14–15.36]), education lower or equal to high school level (OR = 4.28 [95%CI, 1.28–14.27]) and pre-university level (OR = 3.54 [95%CI, 1.18–10.59]). Among occupational factors tested, working in TB hospital was the only parameter showing the significant association (OR = 1.94 [95%CI, 1.04–3.64]) (Table 3).

## Discussion

Our study demonstrated the high prevalence of latent TB infection estimated by either TST or IGRA positivity among hospital workers and higher risk of infection adjusted for age and other factors in the TB hospital than in a general hospital in Hanoi, Viet Nam. Disagreement between TST and IGRA positivity was largely affected by BCG vaccination history and it was not improved by changing cut-off values of TST. As far as we know, this is the first report on TB infection among HCWs evaluated by IGRA in Southeast Asia.

The overall prevalence of IGRA positivity among HCWs in our study (47.3%) is high and comparable to previous estimates from India, Russia and Georgia (40.0%, 40.8%, and 60.0% respectively) [17]–[19]. Direct comparison is difficult among the studies, because in the previous studies particularly the Russian one, detailed information about age strata has not been shown, which strongly affects the prevalence of TB infection.

The prevalence of TST positivity in our study population was higher than that of IGRA. High false-positive TST reaction due to BCG vaccination given after infancy has been reported [7], [20], especially in individuals less than 40 years old [21]. In fact, the degree of TST/IGRA agreement was low in the group with BCG vaccination in our study, with a significant disproportional increase in TST+/IGRA- over TST-/IGRA+. Furthermore, among parameters tested in our study, BCG history was the only factor to be associated with TST+/IGRA-discordance in univariate and multivariate analysis. Our finding is consistent with the previous reports [10], [11], but different from that of another recent study, where BCG did not account for this discordance [22]. This may be simply due to difference in age of BCG vaccination. Involvement of other unknown factors for the discordance cannot be excluded. Exposure to nontuberculous mycobacterium might be another factor for TST+/IGRA- discordance [11], although nontuberculous mycobacterium is rarely found among smear-positive patients in Viet Nam (unpublished data).

These findings indicate that IGRA is more advantageous than TST with different cut-off values [23]. In Viet Nam, BCG vaccination has been included in the Extended Program of Immunization since 1986 and given within one month after birth. Before this point of time, there were no national guidelines and BCG vaccination was sporadically implemented in several areas and mostly given during childhood. In the heterogeneous background of BCG vaccination, it seems difficult to interpret TST result of the present Vietnamese HCWs even with a higher cut-off value as recommended elsewhere [21], [24]. High agreement level between TST and IGRA in a study from India [17] is probably attributed to the fact that most of their participants were vaccinated at birth.

On the other hand, our study did not find significant associations between BCG history and TST-/IGRA+ discordant results and this finding is consistent with the previous reports [10], [11], [22]. Age was associated with TST-/IGRA+ in one study [10] but this was not confirmed in our study.

The CDC [3] and others [25] recommend performing a two-step TST on all newly employed HCWs to identify HCWs who have had MTB infection. Two-step TST is known to evoke remote infection, weak response by nontuberculous mycobacteria, past BCG or other factors, while IGRA appears to reflect recent rather than remote MTB infection [23]. In our study, the influence of two-step TST on TST/IGRA discordance was not much different from that of one-step TST.

In the TB hospital, the proportion of young HCWs who should lower the overall IGRA positivity was larger than in non-TB hospital. Despite this fact, the IGRA positivity in TB hospital was not low. Occupational factors as well as non-occupational factors have been expected to be associated with latent TB infection. In multivariate analysis using a logistic regression model, working in the TB hospital was significantly associated with twofold increase in odds of TB infection estimated by IGRA. Although previous studies have shown that occupational factors, such as working duration and job category, confer a risk on IGRA positivity [17]–[19], our results did not support their data. Working duration is closely related to age and it was difficult to assess its independent effect on TB infection in our study. On the other hand, our data imply that many staff members pursuing a variety of job in the TB hospital might have a considerable chance of exposure to infectious droplet nuclei. While non-TB hospital is a large hospital including an eleven-story building and located in a site with a large yard, the TB hospital is smaller and more enclosed, where all TB patients and HCWs share the same ambulatory route from the entrance (Figure 1). Personal protective equipment used is mostly surgical mask, which cannot prevent the transmission effectively. This finding suggests that the overall working environment and currently used administrative measures should be reconsidered. The cores of infection control programs should be understood deeply to avoid health-care associated infection of TB or MDR-TB at the worst, when a number of MDR-TB patients are hospitalized for treatment.

Non-occupational risk factors for IGRA positivity have been shown in several studies [17], [19]. Age reflects cumulative exposure to MTB and it was significantly associated with IGRA positivity in our study. Education levels may indicate potential risk of TB infection in non-working environment as well as high risk of nosocomial infection. Although these two risks were not separately assessed in our study design, training may be necessary to increase awareness of prevention of nosocomial infection towards workers with low educational levels. Possible risk of high BMI for TB infection was unexpected, but the effect was highest among all covariates. In fact, high BMI was associated with both TST and IGRA positivity. TB development associated with diabetes accompanied by overweight is known [26], but the relationship between overweight and TB infection itself has not been reported. The results may have been produced by chance. Another independent investigation is necessary to determine whether it can be reproducible.

Among the HCWs with positive results of IGRA, no one took INH for treatment of latent TB infection. INH treatment is a safe and low-cost intervention and recommended by WHO [27] and others [28]. However, in typical health care facilities of TB high burden countries where the risk of TB exposure is high and continuous, HCWs still doubt significance of one-time INH treatment.

Our study has several limitations. Firstly, we were not able to evaluate the risk of infection from non-working environment as mentioned above, although the prevalence of TB infection could be estimated roughly from the annual risk of TB infection based on the TST surveys using a formula recommended elsewhere [29]. In Viet Nam, the infection rate was too different between areas to estimate it (data not shown). Secondly, we did not measure HIV infection in our study population. According to the data from a household survey in Ho Chi Minh city, estimated prevalence of HIV infection there is 0.7% in 2005 [30]. We assume that the prevalence is lower in Hanoi. Thirdly, it was not possible to identify the cause of indeterminate cases of IGRA. We re-performed ELISA for all preserved plasma samples with indeterminate results and obtained completely the same results. In addition, we have paid careful attention to maintain the high quality of this test [16]. All of the indeterminate cases showed low response to both TB antigen and mitogen and the pattern of TST measurements in IGRA indeterminate cases was similar to that of IGRA negative cases. For this reason, while calculating the IGRA positivity we did not include indeterminate cases in the numerator but did include them in the denominator, although our results might have underestimated the true proportion.

In conclusion, there is a potential high risk of TB infection among HCWs, particularly those working in TB health facilities in a TB high burden country. Prompt attention is necessary to prevent TB infection among HCWs, preparing for recent spread of MDR-TB in resource-limited settings. For this purpose, IGRA seems appropriate to estimate latent TB infection accurately, contributing to improve infection control strategy especially for young vulnerable HCWs who have heterogeneous history of BCG vaccination after birth.

## Supporting Information

Table S1 Characteristics of population studied. TB: Tuberculosis. *Others mainly consist of administrative staff and pharmacists.
(0.12 MB DOC)
