# Supplementary material for: Prevalence and Risk Factors for Tuberculosis Infection among Hospital Workers in Hanoi, Viet Nam
Source: PLoS One. 2009 Aug 27;4(8):e6798. doi: 10.1371/journal.pone.0006798 (PMC2728839; doi:10.1371/journal.pone.0006798)
Supplement: Table S1 — Characteristics of population studied. TB: Tuberculosis. *Others mainly consist of administrative staff and pharmacists. (0.12 MB DOC) [file pone.0006798.s001.doc]

**Table S1. Characteristics of population studied.**

| Characteristics |  | Total  (N = 300) |  |  | Non-TB hospital  (N = 150) |  |  | TB hospital  (N = 150) |  |
| --- | --- | --- | --- | --- | --- | --- | --- | --- | --- |
|  |  | n | (%) |  | n | (%) |  | n | (%) |
| Years of age |  |  |  |  |  |  |  |  |  |
|  | 20 - 29 | 81 | (27.0) |  | 26 | (17.3) |  | 55 | (36.7) |
|  | 30 - 39 | 61 | (20.3) |  | 13 | (8.7) |  | 48 | (32.0) |
|  | 40 - 49 | 126 | (42.0) |  | 87 | (58.0) |  | 39 | (26.0) |
|  | ≥ 50 | 32 | (10.7) |  | 24 | (16.0) |  | 8 | (5.3) |
| Sex |  |  |  |  |  |  |  |  |  |
|  | Male | 76 | (25.3) |  | 26 | (17.3) |  | 50 | (33.3) |
|  | Female | 224 | (74.7) |  | 124 | (82.7) |  | 100 | (66.7) |
| Body mass index (BMI) |  |  |  |  |  |  |  |  |  |
|  | < 18.5 | 26 | (8.7) |  | 11 | (7.3) |  | 15 | (10.0) |
|  | 18.5 ≤ < 25.0 | 251 | (83.7) |  | 123 | (82.0) |  | 128 | (85.3) |
|  | 25.0 ≤ | 23 | (7.7) |  | 16 | (10.7) |  | 7 | (4.7) |
| Education level |  |  |  |  |  |  |  |  |  |
|  | High school and lower | 41 | (13.7) |  | 22 | (14.7) |  | 19 | (12.7) |
|  | Primary, secondary and pre-university | 160 | (53.3) |  | 80 | (53.3) |  | 80 | (53.3) |
|  | University and higher | 99 | (33.0) |  | 48 | (32.0) |  | 51 | (34.0) |
| Self-reported history of BCG vaccination |  |  |  |  |  |  |  |  |  |
|  | No BCG history | 118 | (39.3) |  | 59 | (39.3) |  | 59 | (39.3) |
|  | BCG history | 112 | (37.3) |  | 55 | (36.7) |  | 57 | (38.0) |
|  | Unknown | 70 | (23.3) |  | 36 | (24.0) |  | 34 | (22.7) |
| History of diagnosis as TB |  |  |  |  |  |  |  |  |  |
|  | No | 289 | (96.3) |  | 143 | (95.3) |  | 146 | (97.3) |
|  | Yes | 10 | (3.3) |  | 7 | (4.7) |  | 3 | (2.0) |
|  | Unknown | 1 | (0.3) |  | 0 | (0.0) |  | 1 | (0.7) |
| History of TB treatment |  |  |  |  |  |  |  |  |  |
|  | No | 288 | (96.0) |  | 142 | (94.7) |  | 146 | (97.3) |
|  | Yes | 11 | (3.7) |  | 8 | (5.3) |  | 3 | (2.0) |
|  | Unknown | 1 | (0.3) |  | 0 | (0.0) |  | 1 | (0.7) |
| Self-reported TB exposure in working environment |  |  |  |  |  |  |  |  |  |
|  | Never | 38 | (12.7) |  | 31 | (20.7) |  | 7 | (4.7) |
|  | Rarely | 35 | (11.7) |  | 22 | (14.7) |  | 13 | (8.7) |
|  | Occasionally | 93 | (31.0) |  | 64 | (42.7) |  | 29 | (19.3) |
|  | Frequently | 130 | (43.3) |  | 29 | (19.3) |  | 101 | (67.3) |
|  | Unknown | 4 | (1.3) |  | 4 | (2.7) |  | 0 | (0.0) |
| Household contact with active TB patient (present time) |  |  |  |  |  |  |  |  |  |
|  | No | 296 | (98.7) |  | 148 | (98.7) |  | 148 | (98.7) |
|  | Yes | 4 | (1.3) |  | 2 | (1.3) |  | 2 | (1.3) |
| Household contact with active TB patient (past history) |  |  |  |  |  |  |  |  |  |
|  | No | 275 | (91.7) |  | 132 | (88.0) |  | 143 | (95.3) |
|  | Yes | 25 | (8.3) |  | 18 | (12.0) |  | 7 | (4.7) |
| Job |  |  |  |  |  |  |  |  |  |
|  | Medical doctor | 70 | (23.3) |  | 37 | (24.7) |  | 33 | (22.0) |
|  | Nurse | 115 | (38.3) |  | 58 | (38.7) |  | 57 | (38.0) |
|  | Technician | 29 | (9.7) |  | 14 | (9.3) |  | 15 | (10.0) |
|  | Others* | 86 | (28.7) |  | 41 | (27.3) |  | 45 | (30.0) |
| Current working area |  |  |  |  |  |  |  |  |  |
|  | Out-patient department | 19 | (6.3) |  | 10 | (6.7) |  | 9 | (6.0) |
|  | TB ward | 57 | (19.0) |  | 0 | (0.0) |  | 57 | (38.0) |
|  | Non-TB ward | 71 | (23.7) |  | 67 | (44.7) |  | 4 | (2.7) |
|  | TB bacteriology laboratory | 16 | (5.3) |  | 0 | (0.0) |  | 16 | (10.7) |
|  | Non-TB bacteriology laboratory | 4 | (1.3) |  | 4 | (2.7) |  | 0 | (0.0) |
|  | Non-bacteriology laboratory | 20 | (6.7) |  | 16 | (10.7) |  | 4 | (2.7) |
|  | Bronchoscopy unit | 3 | (1.0) |  | 1 | (0.7) |  | 2 | (1.3) |
|  | Administration | 81 | (27.0) |  | 37 | (24.7) |  | 44 | (29.3) |
|  | Others | 29 | (9.7) |  | 15 | (10.0) |  | 14 | (9.3) |
| Mask use |  |  |  |  |  |  |  |  |  |
|  | Never or rarely | 64 | (21.3) |  | 42 | (28.0) |  | 22 | (14.7) |
|  | Occasionally | 94 | (31.3) |  | 50 | (33.3) |  | 44 | (29.3) |
|  | Frequently | 142 | (47.3) |  | 58 | (38.7) |  | 84 | (56.0) |
| Years served in the health care profession |  |  |  |  |  |  |  |  |  |
|  | < 2 | 40 | (13.3) |  | 9 | (6.0) |  | 31 | (20.7) |
|  | 2 ≤ < 5 | 53 | (17.7) |  | 22 | (14.7) |  | 31 | (20.7) |
|  | 5 ≤ < 10 | 48 | (16.0) |  | 9 | (6.0) |  | 39 | (26.0) |
|  | 10 ≤ | 159 | (53.0) |  | 110 | (73.3) |  | 49 | (32.7) |

TB: Tuberculosis.

*Others mainly consist of administrative staff and pharmacists.
